# Insulin-Like Growth Factor System in Cancer: Novel Targeted Therapies

**DOI:** 10.1155/2015/538019

**Published:** 2015-03-19

**Authors:** Varsha P. Brahmkhatri, Chinmayi Prasanna, Hanudatta S. Atreya

**Affiliations:** ^1^NMR Research Centre, Indian Institute of Science, Bangalore 560012, India; ^2^Solid State and Structural Chemistry Unit, Indian Institute of Science, Bangalore 560012, India

## Abstract

Insulin-like growth factors (IGFs) are essential for growth and survival that suppress apoptosis and promote cell cycle progression, angiogenesis, and metastatic activities in various cancers. The IGFs actions are mediated through the IGF-1 receptor that is involved in cell transformation induced by tumour. These effects depend on the bioavailability of IGFs, which is regulated by IGF binding proteins (IGFBPs). We describe here the role of the IGF system in cancer, proposing new strategies targeting this system. We have attempted to expand the general viewpoint on IGF-1R, its inhibitors, potential limitations of IGF-1R, antibodies and tyrosine kinase inhibitors, and IGFBP actions. This review discusses the emerging view that blocking IGF via IGFBP is a better option than blocking IGF receptors. This can lead to the development of novel cancer therapies.

## 1. Introduction

Insulin-like growth factor (IGF) is a natural growth hormone and plays crucial role in normal growth and development. The IGF family is comprised of insulin and two factors similar to insulin termed IGF-1 and IGF-2. These factors directly regulate cellular functions by interacting with specific cell surface receptors and activating various intracellular signalling cascades. The cellular responses to the IGFs are mediated primarily by the IGF-1 receptor. The IGF-1 receptor is a member of the family of tyrosine kinase growth factor receptors.

IGFs actions are regulated by six soluble IGF binding proteins (IGFBPs) and IGFBP proteases. The IGFBPs comprise a superfamily of six proteins (IGFBP-1-6) that bind to IGFs with high affinity and specificity and a family of IGFBP-related proteins (IGFBP-rPs), which are structurally similar to the IGFBPs but bind IGFs with much lower affinity.

**Figure 1:**
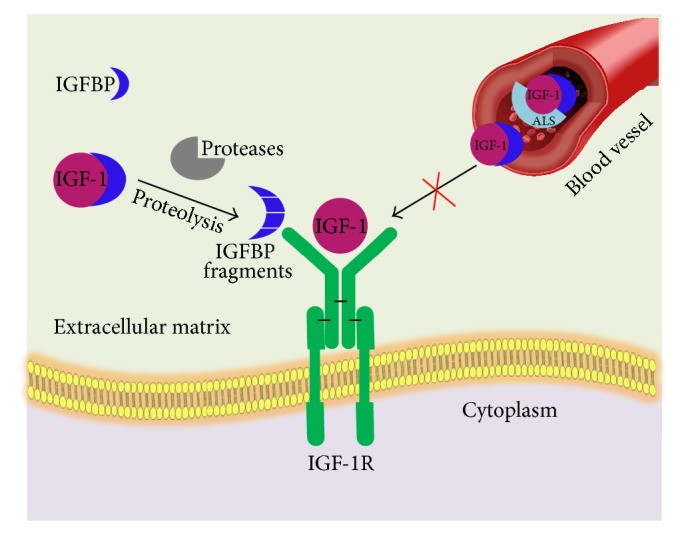
The IGF axis: circulating IGFs are protected from degradation by forming complex with IGFBPs. IGFs, apart from their local functioning in an autocrine or a paracrine manner, enter the bloodstream, where they exist as binary complexes with each IGFBP. In addition, ternary complex also exists when the binary complexes with IGFBP-3 or IGFBP-5 interact with the acid labile subunit (ALS). After dissociation of ternary complex, the binary complexes of IGFBP-IGF are removed from the circulation and cross the endothelium to reach the target tissues and to interact with cell surface receptors.

IGF-1 circulates in relatively high concentrations in plasma, approximately 150–400 ng per mL, where it mostly exists as the protein-bound form. The free ligand concentration is very little that is less than 1% [1]. IGFs in circulation are protected from degradation by forming a complex with a family of high affinity IGF binding proteins (IGFBPs) [2]. IGFBP-3 is the most abundant IGF binding protein in the blood stream followed by IGFBP-2, which is produced in the liver. Most of the circulating IGF-1 and IGF-2 are associated with a high molecular weight complex ~150 kDa consisting of IGFBP-3 and the acid labile subunit (ALS) [2]. Once the ternary complex dissociates, the binary complexes of IGFBP-IGF are removed from the circulation and cross the endothelium to reach the target tissues and to interact with cell surface receptors (Figure 1). In the tissues, IGFBPs may inhibit the interaction of the IGFs with their receptors, as the IGFBPs have a higher affinity for the IGFs than the receptors. In some cases, IGFBPs can enhance IGF action in the local microenvironment by acting as a reservoir that can slowly release the ligands. In addition, some IGFBPs can have IGF-independent effects on cells [2].

The IGFs are signalling proteins (~7.5 kDa) whose actions are mediated by the IGF-1R, and access to the receptor is regulated by the IGFBPs, which vary in size (~22–31 kDa) and share overall sequence and structural homology with each other. The IGFBPs bind strongly to IGFs (*K*_D_ ~ 300–700 pM) and inhibit the action of IGFs by blocking their access to the receptors. Proteolysis of the IGFBPs dissociates IGFs from the complex, enabling them to bind and activate the cell surface receptors. Deregulation of IGF-1R signalling has been noted to contribute to a variety of diseases including diabetic retinopathy [3], diabetic nephropathy [4], age-related macular degeneration [5], cardiovascular disease, and aging and in a variety of cancers [5].

IGF system is gaining tremendous interest over the last decade because it plays an important role in cancer. The current treatment options for cancer have shifted more towards the targeted therapies [6, 7] rather than the traditional chemotherapy. Many strategies have been exploited to target tumours. The most commonly used strategy is engineered antibodies or antibody fragments [8]. Though monoclonal antibodies are very selective, poor penetration inside the tumours and high production cost hinder their usage as therapeutic agents [9]. Current therapeutics targeting the IGF-signalling pathways focus on blocking IGF-1R, directly, and/or its downstream effectors [10]. However, a potential drawback of such approaches is the resulting adverse side effects or toxicities due to its interference with the insulin pathway. As a more efficacious alternative, we propose that IGFBPs can be developed as IGF-antagonist based cancer therapeutics serving to block the IGF-1R, mediated tumour progression. Notably, the IGFBPs do not bind insulin and thus do not interfere with insulin-insulin receptor interactions.

In the current paper, we will provide a brief overview on IGF system and discuss some literature and experimental data reported to demonstrate the role of IGF system in cancer and development of new targeted anticancer therapies. Because it is not possible to provide a complete coverage of all published papers dealing with IGF system, we have mainly focused on different strategies targeting IGF system in cancer and attempted to provide an overview on IGF system including IGF-1R, its inhibitors and potential limitations of IGF-1R, antibodies and tyrosine kinase inhibitors, IGFBP actions, and blocking IGF via IGFBP (which is better option than blocking IGF receptors) leading to development of novel cancer therapies.

## 2. Discovery/History of IGF System

The first member of IGF family to be identified was insulin, with subsequent investigation resulting in the elucidation of its role in glucose metabolism and its implication in the aetiology of diabetes mellitus. This discovery effected an explosion in the investigation of the structure, function, and mechanisms of action of insulin. The enormous interest in this molecule resulted in the concession of three Nobel Prizes for the investigation of insulin: in 1923 for the discovery of its capacity to treat diabetes by Frederick Banting and J. J. Macleod [11], in 1958 for the first sequence of a protein by Frederick Sanger [12], and in 1963 for the first determination of the three-dimensional structure of a protein by Dorothy Hodgkin [13]. Hence, the investigation of insulin has been a pioneer in many scientific fields. Later, the IGFs were discovered and found to be intricately involved in embryonic development and postnatal growth.

The existence of the IGFs was first proposed by Salmon and Daughaday in 1957, on the basis of studies indicating that growth hormone (GH) did not directly stimulate the incorporation of sulfate into cartilage but rather acted through a serum factor [14]. In the original study by Salmon and Daughaday, ^35^S-labeled amino acid was incorporated into cartilage explants and was used as a surrogate for growth. The serum of normal rats induced ^35^S-amino acid incorporation into cartilage, but not serum from hypophysectomized rats. However, serum from hypophysectomized rats treated with GH yielded serum that allowed for ^35^S-amino acid incorporation, indicating that a second messenger was necessary for GH signalling. This factor was originally termed sulfation factor, then somatomedin, and, ultimately, insulin-like growth factor-1 and insulin-like growth factor-2. IGF-1 was not purified and characterized until more than two decades later [15]. The terminology “insulin-like” was used because these factors are able to stimulate glucose uptake into fat cells and muscle, and, indeed, both IGF-1 and IGF-2 show approximately 50% homology with insulin [15, 16].

Subsequent investigation demonstrated that GH, after binding to its transmembrane receptor, initiates a signalling cascade leading to transcriptional regulation of IGF-1 and related genes. It was originally thought that systemic growth was promoted by GH acting mainly on the liver to stimulate IGF-1 production, which then reached target tissues via the circulation to activate mechanisms involved in tissue proliferation, growth, and metabolism. It is now evident that not only does GH have independent actions that do not involve IGF-1 production [17], but IGF-1 synthesis occurs in many tissues under the control of a variety of local and circulating factors, which may or may not include GH [18–21]. Furthermore, this local production of IGF-1 may be directly responsible for the growth promoting effects of GH, rather than the circulating growth factor [22].

### 2.1. IGF-1 Synthesis and Secretion

**Figure 2:**
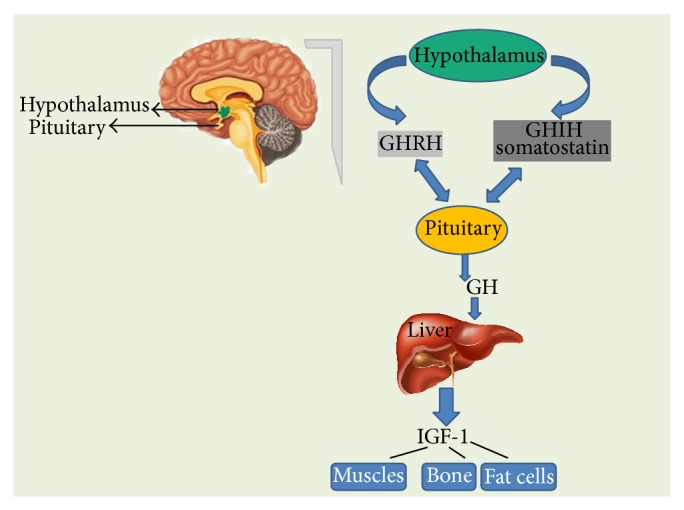
Growth hormone-releasing hormone (GHRH) is a hormone, produced by the hypothalamus which stimulates the pituitary gland to produce GH. Somatostatin secreted by the cells of hypothalamus and also by the cells of stomach, intestine, and pancreas that inhibits GH production. When pituitary secretes GH into the bloodstream, it results in the production of IGF-1 in the liver. IGF-1 is the factor that actually causes the growth of bones and other tissues of the body. It also plays an important role in signalling the pituitary to reduce GH production.

IGF functions as both a circulating hormone and as a tissue growth factor. Liver is the production house for the most circulating IGFs that are subject to both hormonal and nutritional factors. Growth hormone (GH), which is produced in the pituitary gland under the control of the hypothalamic factors, stimulates IGF-1 production (Figure 2).

The insulin like growth factor binding proteins (IGFBPs) are also synthesized in the liver. The IGF ligands in addition to the IGFBPs are delivered in an endocrine manner through the circulation from the liver to act in IGF-responsive tissues. IGFs and IGFBPs are also produced in other organs where autocrine or paracrine mechanisms take place, frequently involving interactions between stromal and epithelial cell populations [23].

### 2.2. Autocrine and Paracrine Actions of IGF

The insulin-like growth factors play a major role in regulating cell proliferation and inhibiting apoptosis. The IGFs are expressed ubiquitously and act in an autocrine/paracrine manner through binding to the IGF-1 receptor (IGF-1R). The bioavailability of IGF in tissues is determined by both local and systemic factors. The local factors include the levels of receptors that are expressed, various IGF binding proteins (IGFBPs), and IGFBP proteases. The systemic factors involved are mainly those that regulate the circulating levels of IGFs, such as growth hormone (GH) and various nutritional factors. Studies in cultured cells have demonstrated that the IGF-1R is frequently overexpressed in cancer cell lines.

The IGFs are not stored within cells of a specific tissue but are present at very high levels throughout the body. They circulate at total concentrations approximately 1000 times higher than those of most peptide hormones and although tissue levels are somewhat lower, they are still present in vast excess compared to that required for maximal cellular stimulation. These high levels are maintained due to their association with the IGFBPs, which dramatically slow their clearance. The IGFBPs bind the IGFs with greater affinity than their cell surface receptors, enabling them to tightly control tissue activity. The IGFBP proteases modify the IGFBPs, lowering the affinity with which they bind IGFs. In the tissues, the IGFs are regulators of cell survival, growth, metabolism, and differentiated function; the complex system confers specificity on these actions.

### 2.3. Evidence for Paracrine/Autocrine IGF-1 Actions from Studies of Transgenic Mice

The most convincing evidence of local IGF-1 actions comes from lines of transgenic (Tg) mice made to overexpress IGF-1 in specific tissues, for example, brain, mammary gland, and muscle. Each of these Tg mouse models exhibits specific overgrowth in the organ or tissue of IGF-1 overexpression, and none has an alteration in circulating IGF-1 levels. Reports of such mouse models are summarized in Table 1. In every model studied biologic actions in the organ of IGF-1 transgene expression have been demonstrated. IGF-1, therefore, can exert local in vivo actions.

Other experiments that address IGF-1 local actions are the generation of Tg mice that overexpress IGFBPs in specific tissues. Here, the expectation is that these IGFBPs will inhibit the actions of locally expressed IGF-I. Such studies have yielded results consistent with those obtained from studies of site-specific IGF-1 overexpression. An example is the overexpression of rat IGFBP-4 in smooth muscle driven by the regulatory region of the *α*-actin gene [24]. Transgene IGFBP-4 expression results in smooth muscle hypoplasia. The lack of any change in circulating IGFBP-4 or IGF-1 and the restriction of hypoplasia to smooth muscle argue for the inhibition of IGF-1 growth promoting effects on smooth muscle. Alternative, but unlikely, interpretations are that IGFBP-4 inhibited the actions of IGF-1 derived from the circulation and/or that IGFBP-4 inhibits growth by mechanisms independent of IGF-I. Other Tg mouse models have yielded consistent results. For example, a number of lines of IGFBP-1 Tg mice exhibit organ growth retardation that appears due to the capacity of IGFBP-1 to inhibit IGF activity in specific tissues, for example, in brain [25–27].

## 3. IGF Receptors (IGF-Rs)

**Figure 3:**
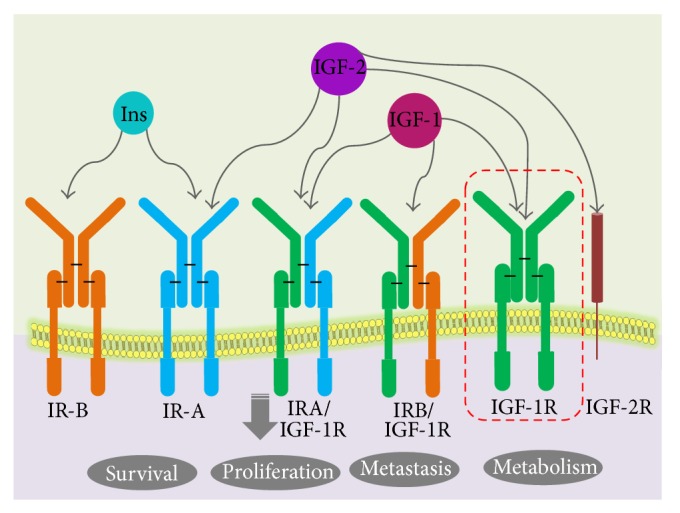
IGF receptor signalling: IGF-1R is a tetramer of two identical *α*-subunits and two identical *β*-subunits. IGF-1R and IR can hybridize to form a heterodimer composed of one *α*-subunit and one *β*-subunit of each receptor. Formation of hybrid receptors is explained with different colour code scheme. IGF-IIR, the mannose-6-phosphate (M6P) receptor, has high affinity for binding the IGF-II ligand but is a nonsignalling receptor. The biological activities of the IGF ligands are mediated by IGF-IR, but the IGF-IIR is considered to function as a “sink” that controls the local bioavailability of IGF ligands for binding to the IGF-IR.

The IGF system comprises two main receptors (IGF-1R and IGF-2R). Both IGFIR and IGF-2R are transmembrane glycoproteins that differ completely in their structure and function [19–21, 28–31]. IGF-1R is a tetramer which comprised two equal *α*-subunits and two equal *β*-subunits [28, 29, 32]. IGF-1R resembles the insulin receptor at structural level, with 60% homology. IGFs and insulin are proficient to cross-bind to each other's receptor, although with much weaker binding affinity than that for the preferred ligand [33, 34]. IGF-1R and IR can hybridize to form a heterodimer composed of one *α*-subunit and one *β*-subunit of each receptor [28, 30] as shown in Figure 3. The amount of insulin/IGF-1 hybrid receptor varies significantly from tissue to tissue. Since its binding affinity for IGF-1 is higher than that for insulin, the receptor is thought to function principally as an IGF-1 receptor, but its biologic significance remains mostly unidentified.

The postreceptor signal transduction events include phosphorylation of insulin receptor substrate (IRS) family of proteins and activation of phosphatidylinositol-3 (PI-3) and mitogen-activated protein kinases (MAPK) [19, 35]. This will result in a myriad of events, including the upregulation of cyclin D1 leading to the phosphorylation of retinoblastoma protein and expression of downstream target genes such as cyclin E [36, 37]. Moreover, IGF-1R activation downregulates the cell-cycle suppressors like PTEN [38, 39], indicating that multiple pathways are involved in producing its mitogenic effect. Activated IRSs trigger the activation of two intracellular signaling networks: Ras/Raf/Mek/Erk and PI3K pathways. The first one is mainly involved in mediating the mitogenic effect of insulin and IGFs, while the PI3K pathway, via Akt, mediates both metabolic and cell growth responses. The Akt-mediated metabolic effects are induced by the activation of enzymes involved in gluconeogenesis, glucose uptake, protein synthesis, and lipogenesis, whereas the cell growth responses are mainly induced by the mTOR pathway.

IGF-2R is monomeric [29, 40–42], the largest transmembrane receptor that is completely unrelated to the IGF-1R, and insulin receptor (IR). In the extracellular domain of the receptor, three ligand-binding regions are found one for IGF-2 binding and two for proteins containing mannose-6-phosphate (M6P) and the dormant form of transforming growth factor- (TGF-) *β* [30]. Binding of IGF-2R, to TGF-*β*, activates the latter [40, 43]. IGF-2R is also called the IGF-II/M6P receptor as it can bind both IGF-2 and M6P-containing Molecules. The expression of IGF-1R is regulated by steroid hormones and growth factors [29, 32]. Since high IGF-1 levels result in a low levels of IGF-1R, IGFs may act as negative feedback signals to suppress expression of IGF-1R [44, 45]. In contradiction of the effect of IGFs, other growth factors, including basic FGF, PDGF, and EGF, stimulate IGF-1R expression [32, 46, 47]. The expression of IGF-1R is also stimulated by estrogens, glucocorticoids, GH, FSH, luteinizing hormone, and thyroid hormones [28, 32]. On the other hand, tumour suppressor gene products, such as wild type p53 protein and WT1 (Wilms' tumour protein), inhibit expression of IGF-1R [11, 48–50]. IGF-1R levels are also affected by nutrition [13, 51, 52]. Not much is known about the regulation of IGF-2R expression, although some studies [29, 30, 53, 54] have suggested that insulin, IGFs, EGF, and M6P may increase the level of IGF-2R, in the cell membrane. Binding of IGFs to IGF-1R activates the receptor's tyrosine kinase activity, which starts a cascade of reactions among a number of molecules involved in the signal transduction pathway (Figure 3).

IGF-2R acts as a scavenger for circulating IGF-2 uniquely. The extracellular domain of the receptor disassociates upon proteolytic cleavage, from the cell membrane as a soluble fragment, circulating in the blood with the ability to bind to IGF-2 and facilitate its degradation [55–60]. These receptors, additionally to the IGFBPs, provide an extra control on the circulating levels of IGF-II.

## 4. Insulin-Like Growth Factor Binding Proteins (IGFBPs)

The insulin-like growth factor binding proteins (IGFBPs) were originally discovered while purifying IGF-1 from serum [61, 62]. The insulin-like growth factors (IGFs) are present in extracellular fluids bound to high affinity carrier proteins (Table 2). Six forms of IGF binding proteins (IGFBPs) have been cloned and their complete sequences have been obtained [63].

IGFBPs have three domains. Human IGFBPs 1–6 each contain 216–289 amino acids organized into three domains of approximately equal size, with the conserved N- and C-domains being joined by a “linker” L-domain [2, 64]. IGFBPs 1–5 have 18 conserved cysteines, whereas IGFBP-6 has 16 [2, 65]. The N-domains of IGFBPs 1–5 contain six disulfides and share a conserved GCGCC motif; IGFBP-6 shares all of these except the two adjacent cysteines in this motif. Therefore, the first three N-terminal disulfide linkages of IGFBP-6 differ from those of IGFBP-1 and, by implication, the other IGFBPs [65]. By contrast, the remaining N-domain disulfides and all three C-domain disulfides are probably conserved in all IGFBPs.

**Figure 4:**
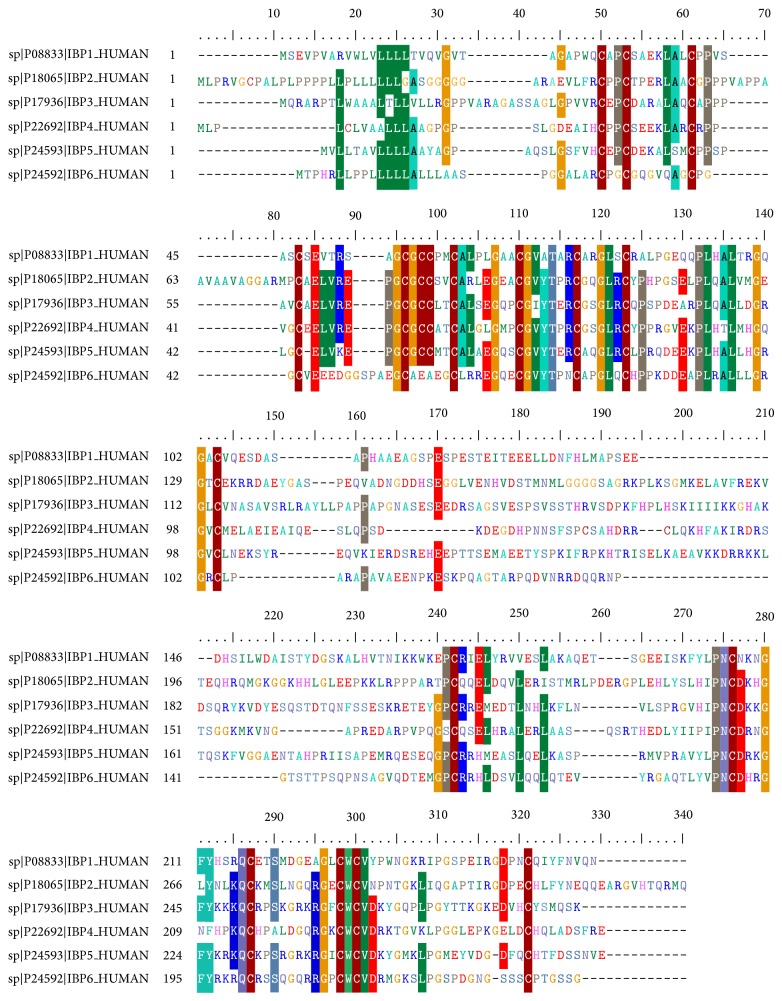
Amino acid sequence alignment of human IGFBP-1 to IGFBP-6. Alignment was performed using the ClustalW program. Small gaps were introduced to optimize alignment.

The sequence alignment of IGFBPs 1–6 is depicted in Figure 4, where the N-domains of IGFBP 1–5 contain six disulfides and share a conserved GCGCC motif; IGFBP-6 shares all of these except the two adjacent cysteines in this motif. The C-domains are known to share the highly conserved CWCV motif. But the central domains do not contain any cysteines and exhibit little homology.

The six IGF binding proteins are unrelated to the cell surface receptors but are structurally very closely related to each other, although they are each products of distinct genes and they all have very distinct functional properties.  Table 3 summarizes the results of inhibiting IGFBPs activity and their role in cancer.

### 4.1. IGFBP Proteases

Ever since the discovery of IGFBP-3 protease in seminal plasma [66] and human pregnancy serum [67], IGFBP proteases have been known to be present in various body fluids [68]. IGFBP proteases belong to a superfamily of proteases with specificity towards IGFBPs, thereby regulating the action of IGFBPs. These proteases are prime factors in modulating the levels of IGFBPs and ultimately the bioactivity and downstream actions of IGFs [69].

IGFBP proteases broadly fall into three major super families—serine proteinases (kallikrein-like serine protease), matrix metalloproteinases (MMPs), and cathepsins [70, 71]. The work of Cohen et al. demonstrating the significance of IGFBP proteases and a descriptive review by Fowlkes talk miles about their classification [70, 71].  Table 4 summarizes different IGFBP proteases and their target substrates with target sequence specificity.

Prostate specific antigen (PSA), the first IGFBP protease to be discovered in seminal plasma [66] and later on in pregnancy serum [72], is a serine proteinase produced by the prostate gland and is known to degrade IGFBP-3 [66]. *γ*-nerve growth factor (NGF), homologous to PSA, is also known to degrade IGFBP-3 and IGFBPs 4, 5, and 6, thereby enhancing IGF actions. Epidermal growth factor binding protein (EGFBP), human plasma kallikrein (hPK), and renin are relatively poor IGFBP proteases [71].

Matrix metalloproteinases are calcium-dependent zinc-containing endopeptidases, with the capability of degrading several extracellular matrix molecules including collagens, elastins, gelatin, matrix glycoproteins, and proteoglycan [71, 73–75]. These extracellular degrading enzymes are also known to be active against IGFBPs [74]. They were first discovered as IGFBP-3 proteinases in human dermal fibroblasts [74, 76]. These MMPs are known to contribute to the degradation of IGFBPs 1, 2, 3, 4, and 5 known from various scientific studies including a study showing the proteolytic cleavage of IGFPB-1 and IGFBP-2 by MMP-1 in smooth muscle airway cells. [71, 73, 76–78]. Research has shown that MMP-3 and MMP-9 can cleave IGFBP-1. MMP-1 and MMP-3 degraded rhIGFBP-3 to much greater extent than MMP-2 in vitro [74]. ADAM-12, a disintegrin metalloproteinase, is also known to have proteolytic activity against IGFBP-3 [77].

Cathepsins belong to a family of lysosomal proteinases with optimal activity in acidic conditions discovered by their proteolytic activity on IGFBP-3 [70, 71]. Cathepsin D is a well-known IGFBP protease shown to have proteolytic activity against IGFBPs 1–5 in acidified condition [70, 79]. In neutral conditions, their proteolytic activity seems to be unclear.

The central linker domain which is the least conserved region has not been cited to be a part of the IGF binding site for any IGFBPs but is reported to have four major protease cleavage sites in IGFBP-2, determined to be between Tyr103 and Gly104, Leu152 and Ala153, Arg156 and Glu157, and Gln165 and Met166 [80]. A study involving mutation of selected residues of the linker domain of IGFBP-4 led to protease resistivity of IGFBP-4 [81]. This leads to the conclusion that the proteolysis of IGFBPs occurs at specific sites by proteases in unstimulated, homeostatic conditions (e.g., PAPP-A activity in normal cell lines). As the reports suggest the linker domain to be most proteolysis susceptible among the N-, C-, and the linker domain, it acts as the determinant in the release of IGF from IGFBPs. Thus, a detailed understanding of the interaction of L-IGFBP-2 with IGF at atomic level is important. This may help to determine the changes which can be brought about in the linker domain for careful modulation of IGF release, which could in turn prevent unwanted IGF-1R, signalling controlling abnormal cellular growth and proliferation. Alternatively, in conditions where cellular proliferation is desired (e.g., wound healing), control on release of IGF may facilitate IGF mediated cellular growth and proliferation. Thus, a study of the structure of linker domain (L-IGFBP-2) and its interaction with IGF-1 together with the change in dynamics in presence of IGF-1 was studied in our laboratory.

### 4.2. Significance of IGFBP Proteases in Cancer

IGFBP proteases are known to target and degrade IGFBPs to smaller fragments and thus bring down the affinity of IGFBPs to IGFs. This results in IGFs binding to their respective IGF receptors resulting in signalling cell proliferation, growth, and cell migration. Kallikreins have also been employed as biomarkers in cancer [82]. Apart from the significance of proteolysis in regulating the bioavailability of IGFs in tissues and increasing the affinities of IGFs to IGF receptors, this seems to play a significant role in tumour progression and tumour cell survival considering the autocrine-paracrine actions in the IGF axis. Thus, IGFBP proteases have potential clinical implications in cancer research.

A novel approach in this regard is development of mutant IGFBPs lacking the IGFBP protease cleavage sites, rendering them protease resistant. This serves as a potential therapeutic agent as it inhibits IGF signally through IGF receptors. Such studies reported a decade ago where a protease resistant IGFBP-4 was designed and in vivo studies of this protease resistant IGFBP-4 [81] were explored confirming the complete resistance to IGFBP-4 protease indicating that the mutant IGFBP-4 resulted in greater growth inhibition than equivalent levels of native IGFBP-4 demonstrating a role for IGFBP-4 proteolysis in the regulation of IGF-1 action and a potential implication in cancer [81]. In yet another similar in vivo study, protease resistant IGFBP-4 has been shown to block IGF activity, tumour growth, and angiogenesis [83].

In another such recent study, a novel approach has been used to develop protease resistant (PR) and protease resistant/non-matrix-binding (PR/NMB) variants of IGFBP-2 as potential tumour growth inhibitors [84]. They hypothesized that lack of protease and matrix-binding sites render the IGFBP-2 devoid of the ability to promote IGF-dependent action (through release of IGFs to the receptors) and IGF-independent action (through ECM binding). The in vitro and in vivo studies indicate that the mutant IGFBP-2 (lacking a large portion of the central linker domain) is able to inhibit tumour growth possibly by inhibition of angiogenesis. Their studies promise to open up new avenues for better targeting strategies for the effectiveness of cancer treatment in the near future.

### 4.3. IGFBP-Related Proteins (IGFBP-rPs)

The IGFBP superfamily includes 6 members (IGFBP-1 to IGFBP-6) with high affinity for IGF-1 and IGF-2 and 10 IGFBP-related proteins (IGFBP-rP1 to IGFBP-rP10) with low affinity for these ligands. Remarkably, IGFBP-related protein 1 (IGFBP-rP1), also known as insulin-like growth factor binding protein-7 (IGFBP-7) [85], is identified as a secretory and low-affinity IGFBPs. It is distinct from other low-affinity IGFBP-rPs in that it can bind strongly to insulin [86], suggesting that IGFBP-7 is likely to have distinct biological functions from other IGFBPs. IGFBP-related protein 1 (or IGFBP-7) has been found to have an important role in the female reproductive system. It was implicated in human endometrial receptivity, folliculogenesis as well as growth, development, and regression of the corpus luteum in higher mammals [87–89]. Other studies showed that it could induce apoptosis in M12 prostate cancer cell line [90].

Rupp et al. demonstrated that, adding to IGFBP-7 tumour suppressor function, it can promote anchorage-independent growth of malignant mesenchymal cells and of epithelial cells with an EMT-phenotype when IGFBP-7 is expressed by the tumour cells themselves [91]. Expression of IGFBP-7 in tumour-associated fibroblasts can also promote colony formation when epithelial tumour cells are cocultured with IGFBP-7-expressing cancer-associated fibroblasts (CAFs) by secondary paracrine tumour-stroma interactions. Zhu et al. recently reviewed role of insulin-like growth factor binding protein-related protein 1, IGFBP-rP1, in cancer [92]. In many cancers, IGFBP-rP1 acts as a tumour suppressor gene by suppressing proliferation and inducing apoptosis and senescence. However, there are some contradictory data and different opinions; for example, IGFBP-rP1 has been reported as promoting glioma cell growth and migration [93]. It has been recently reported that IGFBP-rP1 could bind to the IGF-1R and block its activation [94].

### 4.4. IGFBP Structure

**Figure 5:**
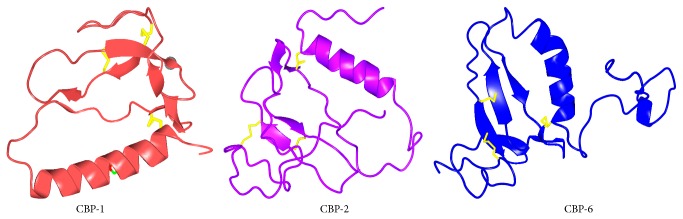
Structures of C-terminal domains of IGFBP-1, IGFBP-2, and IGFBP-6 represented as CBP-1 (**1ZT5**), CBP-2 (**2H7T**), and CBP-6 (**1RMJ**), respectively.

The structural features of IGFBPs, which carry IGFs in the circulation, are very important for understanding their role in normal growth and development as well as in diseases. The insulin-like growth factor binding protein-2, the second most abundant IGFBP in circulation and known to form binary complexes with IGF, is 32 kDa (289 amino acid residues) in size with three distinct regions: the highly conserved N-terminal region (IGFBP homolog domain), the highly conserved C-terminal region with thyroglobulin type 1 repeat [95], and the mid-region known as the linker domain of IGFBP-2 with multiple cleavage sites. The structures of C-terminal domains of IGFBP-1, IGFBP-2, and IGFBP-6 are shown in Figure 5.

Notably, the C-terminal domain contains an arginine-glycine-aspartic acid (RGD) motif which can bind to integrins and take part in cell mediated signaling. The N- and C-terminal domains are cysteine rich and are structured, with both of them having IGF binding properties capable of modulating the IGF/IGF receptor interactions [96]. While some reports have emphasized the importance of the binding of N-terminal domain to IGF by mutagenesis experiments [97] and by iodination protection study [98], others have described the C-terminal region of IGFBP-2 as playing important role in the binding to IGFs by mutagenesis experiments [99, 100] and by nuclear magnetic resonance spectroscopy [101]. Some others emphasize the cooperative role which the N-terminal and the C-terminal domain play in the binding to IGF-1 [102]. The structural aspects of IGFBPs have been recently reviewed by Forbes et al. [103]. The important structural features for interaction of IGFBPs with extracellular matrix and integrins were described. Further, they highlighted the important structural features for binding with IGFs and other partners also.

### 4.5. Structural Studies of Human IGFBP-2 Binding by NMR

While the biological actions of IGF-1-IGFBP-IGF-1R axis have been extensively studied, a complete understanding of IGF-IGFBP interactions on a structural level is lacking. Our objective was to elucidate the mechanistic aspects of IGF-IGFBP interactions at the atomic level in order to develop IGFBPs as cancer therapeutics.

A critical challenge in the structural characterization of full-length IGFBPs has been the difficulty in expressing large amounts of these proteins for NMR/X-ray crystallography analysis. We have developed a method for high-yield expression of full-length recombinant human IGFBP-2 (hIGFBP-2) in* E. coli* [104]. Using a single step purification protocol, we obtain hIGFBP-2 with >95% purity. The protein exists as a monomer at the high concentrations (up to 30 mg/mL) required for structural studies in a single conformation exhibiting a unique intramolecular disulfide-bonding pattern. We have thus, for the first time, obtained high-yield expression of wild type recombinant human IGFBP-2 in* E. coli* and initiated structural characterization of a full-length IGFBP. We are currently studying the molecular interactions of the different domains of hIGFBP-2 with IGF-1, in particular the central flexible domain which is known to play a pivotal role in the protein function and regulation. These are described in the proceeding section.

#### 4.5.1. Study of Nanotubular Structures Formed by a Fragment of IGFBP-2

**Figure 6:**
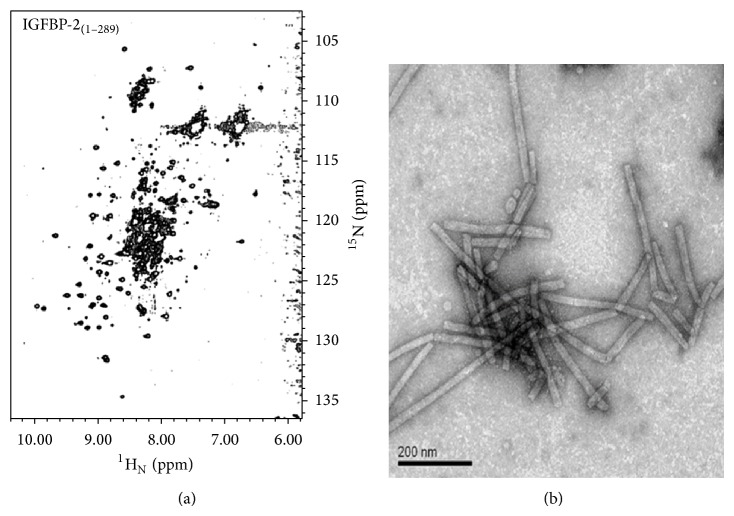
(a) 2D [^15^N-^1^H] HSQC spectrum of purified full-length hIGFBP-2 (1.0 mM; nondeuterated) recorded at a ^1^H resonance frequency of 800 MHz at 285 K. (b) TEM images of (hollow) nanotubular structures formed by the C-terminal fragment of human IGFBP-2.

We recently discovered that the C-terminal fragment of hIGFBP-2 (residues 249–289) self-assembles spontaneously and reversibly into nanotubular structures under nonreducing conditions and remains as a monomer under reducing condition. These nanotubular structures were studied extensively by transmission electron microscopy (TEM), NMR spectroscopy (Figures 6(a) and 6(b)), and circular dichroism (CD) and a mechanism for their formation has been worked out [105].

#### 4.5.2. Biomedical Applications of IGFBP-2 Nanotubes

The presence of an RGD motif in this polypeptide fragment offers avenues for novel biomedical applications. The RGD motif is known to be recognized by integrins. The design of such self-assembling polypeptide fragments containing an RGD motif can be utilized to enhance the efficacy of cancer therapeutics. We have explored the possibility of using these nanotubes for cancer cell imaging. This is based on the idea that, in many cancers, integrins are expressed in large quantities on the cell surface. Thus, IGFBP-2_249–289_ nanotubes can be developed to identify the location of cancer cells through their binding to integrins via the RGD motif. Towards this end, we have carried out cell-adhesion and cell-proliferation assays which have helped to characterize the binding of the nanotubes to integrin via the RGD motif.

## 5. Therapeutic Strategies Targeting IGF System in Cancer 

Therapeutic strategies targeting various components of the IGF system, with varying degree of success, have been developed for treatment of different types of cancer. Description and challenges of each targeting strategy will be enlightened in this section.

### 5.1. Targeting IGF-R: Therapeutic Potential of IGF-Rs in Cancer

IGF-1R activation by tyrosine phosphorylation of *β* subunit results in activation of PI3K/AKT and RAS/MAPK pathways [106, 107] which in turn regulate cell survival and proliferation. IGF axis is tightly regulated under normal physiological conditions maintaining cell homeostasis and growth. Genetic alterations of IGF-1R leading to varying levels of their expression are found to have a link in cancer [108]. These receptors maybe activated in the tumour cells in an unregulated manner. (mutation, chromosomal translocation, abnormal stimulation, and loss of genomic imprinting).

IGF-1R does not solely drive tumour cell proliferation; however, most oncogenes are required in mediating anchorage independent growth given its property to mediate proliferation and cell survival. This is one of the key processes to achieve metastasis among tumour cells [107, 109].

High levels of IGF-1 have been reported in several cases of breast and prostate cancers [110] and since IGF-2 is maternally imprinted [111, 112], loss of this imprinting results in biallelic expression, resulting in increased IGF-2 production and a suspected mechanism of cancer development and progression in many conditions [111, 113–115]. These higher levels of IGF-1 and IGF-2 promote IGF-1R signalling and the consequently activated downstream pathways. Increases in IGF-1R have been shown in different types of cancer, melanoma, and carcinomas [116–118]. Considering disease prognosis, therapeutic approaches based on targeting IGFRs seem to be promising in cancer research.

Another aspect of IGF-R is the formation of IGF-1R/IR hybrids by random association of insulin half-receptor (IR-A) with an IGF half-receptor adding further complexity in receptor targeting strategy [119]. IR isoform (IR-A) is overexpressed in cancer and it is the fetal isoform of IR (while other half is IR-B involved in regulating glucose uptake) and IGF-1R is also overexpressed in cancer. With the overexpression of these receptors, formation of IGF-1R/IR hybrid receptors is expected. These have broad binding specificity as they bind IGF-1, IGF-2, and also insulin [119]. Targeting these hybrid receptors becomes one of the several strategies.

**Figure 7:**
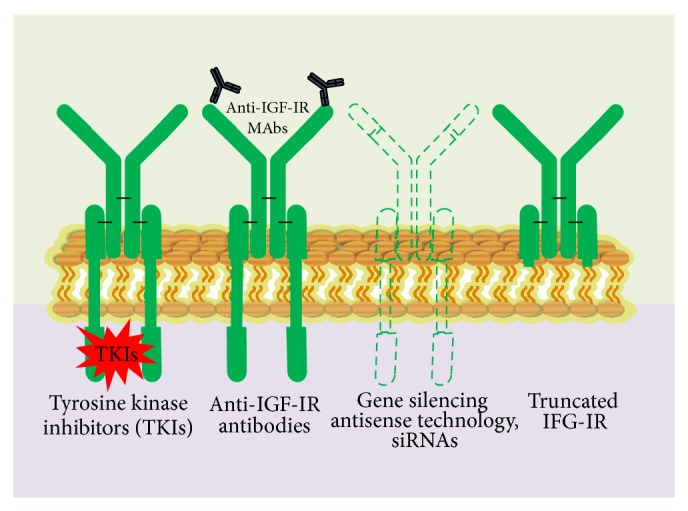
Various strategic approaches to targeting IGF-1R receptors. Small-molecule TKIs, inactivating anti-IGF-1R antibodies, reduction or elimination of IGF-1R, protein expression by blocking IGF-1R, transcription (with triple helix) or translation (antisense technology and siRNA), IGF-1R, and mutants lacking beta-subunits (dominant-negative receptors).

There are several approaches of targeting IGF-R till date, namely, small molecule tyrosine kinase inhibitors (TKIs), anti-IGF-1R antibodies, and molecular agents such as antisense and small interfering RNAs (si-RNAs) [107, 120] (Figure 7).

While a lot is known on targeting IGF-Rs through TKIs and anti-IGF-1R antibodies and there are detailed multiple reviews on their targeting strategies [108, 120–129], little is known on targeting the former using antisense technology and si-RNAs. Tables 5 and 6 summarize few of the several different TKIs and anti-IGF-1Rs studied.

Recent advancements in this approach show us that it is possible to genetically target IGF-Rs. Adenoviruses expressing antisense IGF-1R and truncated IGF-1R, nonviral vectors expressing truncated IGF-1R, were used to successfully block IGF-1R, thereby suppressing tumorigenicity in vitro and in vivo, and also effectively blocked both IGF-1- and IGF-2-induced activation of Akt-1.

Studies in which small interfering RNAs (siRNAs) induce potent IGF-1R gene silencing without affecting the insulin receptor demonstrate that siRNAs block IGF signalling, thereby enhancing radio and chemosensitivity and paving yet another way of therapeutic potential, and may in future generate nucleic-acid-based therapeutics [125, 130]. The efficacy of IGF-1R targeting in the clinics depends on major factors such as the role of IFGR in itself in the tumours, inhibition potential of siRNAs and antisense therapies in vivo, and compensation of other signalling pathways due to IGFR loss [130].

These studies also prove the potential genetic blockade studies of IGF-1R and its efficacy and prognosis in several malignancies, lung, colon, and pancreatic carcinoma [131, 132]. Such antisense and dominant negative strategies (truncated) also enhance tumour cell chemosensitivity (effective chemo- and radiotherapy induced apoptosis). One more prominent feature is the immune protection induced by tumour cells killed in vivo by IGF-1R-antisense technique. Major drawback is that antisense agents cause adequate IGF-1R downregulation and also affect insulin receptor.

Cotargeting IGF-Rs along with other tumour promoting pathways is yet another way to effectively overcome the limitations of resistance to conventional chemo- and endocrine therapy to single agent targets discussed in previous sections as cross talk between IFG-R and RTKs/steroid hormones is known to promote tumorigenesis. IGF-1R is known to interact with several pathways and molecules, receptor tyrosine kinases (RTKs), including insulin receptor (IR), epidermal growth factor receptor (EGFR), vascular endothelial growth factor receptor (VEGFR), mesenchymal-epithelial transition factor (MET), platelet-derived growth factor receptor (PDGFR), and fibroblast growth factor receptor (FGFR), and steroid hormones, including estrogen receptors alpha and beta, androgen receptor (AR), and progesterone receptor (PR). This novel approach pertains to cross talk cotargeting [133]. Examples of such a targeting strategy include monoclonal antibodies and small molecule tyrosine kinase inhibitors, in combination or cotargeting IGF-1R and EGFR receptors [123, 134, 135], where simultaneously both receptors are targeted making it a promising novel approach. In a recent study, cotargeting the IGF system and HIF-1 (hypoxia-inducible factor-1) has been shown to inhibit the migration and invasion by breast cancer cells [136], indicating that ligand-targeting compounds, or coinhibition of the IGF and HIF-1 systems, may prevent activation of compensatory signalling (due to cross talks), thereby providing a valuable and novel addition to IGF-1R inhibitor-based therapies [136].

IGF-2R deserves a mention since studies implicate that the mannose 6-phosphate/insulin-like growth factor-II receptor (M6P/IGF-2R) functions in the intracellular trafficking of lysosomal enzymes, the activation of the potent growth inhibition transforming growth factor beta 2, and the degradation of IGF2 (which are overexpressed in tumours). Studies have shown that M6P/IGF-2R gene functions as a tumour suppressor in human liver carcinogenesis [137].

### 5.2. Targeting IGFs: Therapeutic Potential of IGFs in Cancer

The insulin-like growth factors (IGFs), IGF-1 and IGF-2, are ligands that bind to IGF receptor (IGF-1R,) and regulate cancer cell proliferation, survival, and metastasis. Since IGF axis is involved in regulating cell metastasis, the pathway plays a significant role in cancer cell metastasis and proliferation and many studies over a couple of decades have tried to establish the relationship between serum IGF levels and cancer risk.

Many experiments demonstrate the increase in neoplastic cell proliferation with increasing IGF-1 concentration [138]. Various human epidemiological studies describe the correlation between circulating levels of IGF-1 coupled with IGFBPs and the risk of developing various cancers, lung, colon, breast, and prostrate [139–143]. Circulating IGF-1 levels play a significant role as a risk factor in the onset and development of mammary tumours in breast cancer [144]. In vivo studies suggest that carcinogenesis and cancer progression are influenced by germ line variation of genes encoding signalling molecules in the GH-IGF-1 axis and these mutations are often associated with genetic manipulations [144] and low IGF-1 levels; thus, tumour growth is influenced by IGF-1 physiology [145]. Yet the connection between circulating IGF-1 levels and cancer risk remains inadequately hidden. Two contradictory hypotheses on relationship between IGF-1 and cancer risk are underlined by Pollak [146].

Firstly, if a cell at risk is considered (e.g., somatic cell mutations lead to accumulating DNA damage), IGF bioactivity in the cellular microenvironment influences the fate of the cell survival and evolves to malignant cell lineage or apoptosis in early carcinogenesis. To balance apoptotic cell death and survival of damaged cells might be slightly inclined towards survival in an environment with high IGF levels, and this would favour the appearance of a malignant clone. The fate of such millions of DNA damaged cells is determined every hour, and even a modest influence of higher IGF-1 level on survival probability might lead to an association of circulating level with cancer risk [146]. Secondly, the influence of IGF-1 level on cancer risk is somewhat related to early carcinogenesis. Higher IGF-1 levels facilitate the more rapid proliferation of early cancers to the stage at which they can be clinically detected. Such lesions would be common in all adults, and cancer diagnosis would reflect the probability of these lesions progressing toward a detectable and clinically significant size, with this latter process being influenced by IGF-1 level [146].

Findings in the case of prostate cancer may be consistent with this second hypothesis. This is consistent with the view that the IGF-1 level is more related to the probability of progression of early lesions than to the actual process of early carcinogenesis. According to Pollak, both hypotheses are plausible and are not mutually exclusive; also there is no definitive mechanistic evidence to support either of them [146].

IGF-2 is also a ligand for the IGF-1 receptor and is present in serum at concentrations that are generally higher than IGF-I. IGF-2R serves as a sink to IGF-2R and does not allow the signal transduction of the latter and has the characteristics of a tumour suppressor which is discussed in previous section on targeting IGF-Rs [137].

Several drug candidates that target IGF-1 signalling were found to have antineoplastic activity by using in vitro studies and in vivo models, both as single agents and in combination with currently approved drugs. Several high-affinity antibodies are developed which cross-react with both IGF-1 and IGF-2 and these are at their early developmental stage. MEDI-573 is one such human antibody (fully human) that neutralizes both IGF-1 and IGF-2, thus inhibiting IGF signalling through both the IGF-1R and IR-A pathways. Studies also show that MEDI-573 inhibited the in vivo growth of IGF-I- or IGF-II-driven tumours [147]. Hypophysectomy is also thought to be one of the IGF-1 ligand lowering strategies which was also successfully employed in patients with hormone-responsive breast cancer [148]. Advantage of antiligand approach is that it has the potential to block the action of IGF-2 at the insulin isoform A, without interfering with insulin action. This finding is in view of various cancers where IGF-2 production is autocrine [126].

### 5.3. Targeting IGFBPs: Therapeutic Potential of IGFBPs in Cancer

There is accumulating evidence in the literature stating that IGFBPs can also cause apoptosis in an IGF-independent manner [149] and they can show inhibitory effects towards tumour growth and cancer [150].

Although IGFBPs can prevent IGF from binding to IGF-1R, because of their higher affinity to IGF than the IGF-1R, it can also induce tumour growth and progression in situations where the IGFBP proteases levels are high and/or when IGFBPs interact with ECM. Thus, modifying IGFBP depends on the targeted tissue and the disease state. For example, IGFBP-3 has shown proapoptotic, antiproliferative, and antiangiogenic functions in in vitro tumour models [69, 151]. On the other side, IGFBPs can promote tumour progression in the presence of proteases. IGFBP-2 and IGFBP-5 upregulation in CRPC are a good example of that. In the presence of PSA and other factors affecting the IGF-I/IGFBP-2 and IGFBP-5 binding, it will result in the delivery of the IGFs to the IGF-1R and activation of the downstream signalling 21 pathway, thus helping the progression to castration resistant disease [152, 153]. Recently, Baxter et al. reviewed IGFBPs and their cellular actions beyond their endocrine role in IGF transport [154]. They suggest that IGFBPs can also function in their pericellular and intracellular sections to regulate cell growth and survival. Further they interact with many other proteins including their canonical ligands IGF-1 and IGF-II. Also they have shown that the intracellular functions of IGFBPs in transcriptional regulation, induction of apoptosis, and DNA damage repair which also point to their friendly participation in tumour development, progression, and resistance to treatment.

#### 5.3.1. Cancer Stimulatory/Inhibitory Effects of IGFBPs

*IGFBP-1.* IGFBP-1 has higher IGF-1 binding affinity in various phosphorylated forms than the unphosphorylated protein and is inhibitory to IGF action [155]. An interesting study using IGFBP-1 deficient mice demonstrated that IGFBP-1 can function as a cell survival factor by repressing TGF*β* activation [156], but the relevance of this effort for cancer cell survival is not understood. On the whole there is no specific confirmation that IGFBP-1 stimulates tumour growth or it is extensively a tumour growth inhibitor [157].

*IGFBP-2.* IGFBP-2 overexpression in mice is found to inhibit development of colorectal adenomas by reducing the tumor growth by inhibition of cell proliferation [158]. Further there is significant evidence for a growth promoting effect of IGFBP-2 in many tumour systems, by sequestering IGFs [159]. IGFBP-2 contains an Arg-Gly-Asp motif, but substitution of these amino acid residues did not affect the cell binding of IGFBP-2 [160]. Additionally, this motif interacts with *α*5 integrin and is found to be involved in regulating the effect of IGFBP-2 on glioma cell migration and invasion [161, 162]. 

*IGFBP-3.* IGFBP-3 can function as a cancer suppressor and is downregulated in some cancer tissues. However, growth promotion by IGFBP-3 has been described by several mechanisms, which involve its overlap with other cell signaling systems. Potentiation of IGF-I dependent proliferation by IGFBP-3 that was first described in human skin fibroblasts in 1988 [163], has also been revealed in breast cancer and some other cell types [68, 164–166]. Further in some cases, IGFBP-3 was shown to stimulate IGF-1 action, even for IGF derivatives that have negligible interaction with the binding protein [167], so the consequence is unlikely to involve IGFBP-3 somehow presenting IGFs to their receptor.

In patients with NSCLC, the greatest activation of IGF-1R was observed in tumours that expressed high levels of IGFBP-3 [168], although it is not clear whether this activation was ligand dependent. The high expression levels of both EGFR and IGFBP-3 are seen in tumour tissue compared with normal tissue in case of oesophageal cancer [72]. 

*IGFBP-4.* Cancer inhibitory effects of IGFBP-4 are generally accepted. IGFBP-4 is found to inhibit tumour progression by sequestering IGFs [66], but some reports demonstrate that, in some circumstances, it might suppress cell death [72] or stimulate cell migration. In epithelial ovarian cancer, IGFBP-4 mRNA is found to be highly expressed [170] but has not been shown to be significant for prognosis. 

*IGFBP-5.* In breast cancer models, IGFBP-5 overexpression was strongly tumour inhibitory in vitro and in vivo [171], whereas the opposite effects were observed in some other cancer models, in which IGFBP-5 can stimulate IGF-dependent and IGF-independent cell survival and proliferation [172–175]. In noncancer cell lines, similar effects have been reported [176, 177]. In prostate cancer cells, down regulation of IGFBP-5 inhibited IGF-dependent growth in vitro and in vivo and castration induced upregulation of IGFBP-5 in mice accelerated the development of androgen independence [178]. 

*IGFBP-6.* As recently reviewed [82], IGFBP-6 is also known to have inhibitory effects in cancer by blocking IGF signalling, extraordinarily IGFII, but there is evidence where in some circumstances it may have oncogenic actions stimulating migration [179] and proliferation [70] which is mechanistically stronger than for IGFBP-4. The IGFBP-6 was shown to be involved in cell surface interaction with prohibitin 2, a protein found in the mitochondria and nucleus, as well as in the plasma membrane; thus, it stimulates rhabdomyosarcoma cell migration. IGFBP-6 ligation results in tyrosine-phosphorylation of Prohibitin 2 [180]. Primarily, IGFBP-6 is tumour suppressive [82], but an ultimate link between its activity in vivo remains to be established.

**Figure 8:**
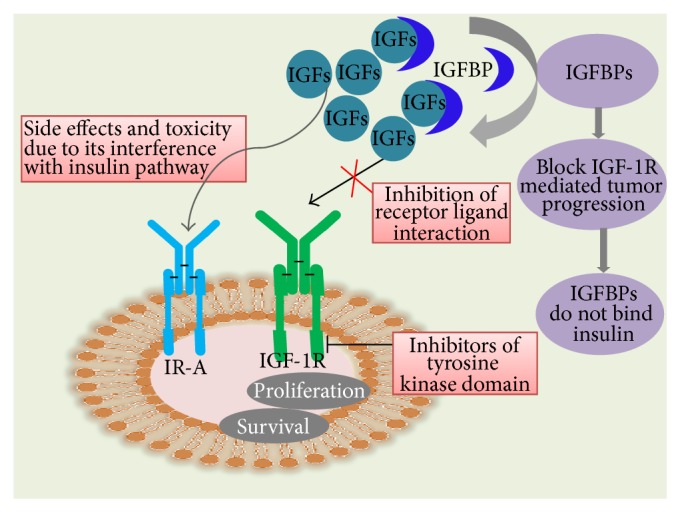
Targeting IGFBPs, a novel strategy in cancer therapeutics. The cancer therapeutics targeting the IGF-signalling pathway focus on blocking IGF-1R, directly, and/or its downstream effect. Drawback of such approaches is the adverse side effects or toxicities due to its interference with the insulin pathway. The more efficacious alternatives, IGFBPs, as IGF-antagonist based cancer therapeutics also contribute to block the IGF-1R, mediated tumour progression. As IGFBPs do not bind insulin, they do not interfere with insulin-insulin receptor interactions.

It is now clear that the IGFBPs have many effects on cell death, via both IGF-dependent and IGF-independent actions. Although the mechanisms underlying these latter actions are only beginning to be understood, it is already clear that they may provide very specific strategies for fine-tuning therapeutic interventions. Current therapeutics targeting the IGF-signalling pathway focus on blocking IGF-1R, directly, and/or its downstream effect. Potential drawback of such approaches is the resulting adverse side effects or toxicities due to its interference with the insulin pathway. As a more efficacious alternative, we propose that IGFBPs can be developed as IGF-antagonist based cancer therapeutics serving to block the IGF-1R mediated tumour progression (Figure 8). The IGFBPs do not bind insulin and thus do not interfere with insulin-insulin receptor interactions.

## 6. Natural Products: Targeting IGF Signalling Pathways

Natural products are known to have medicinal benefits from ancient history. They have been used for the treatment of various diseases and are gaining tremendous importance in the area of drug discovery. These natural product derived phytochemicals have been extensively studied and have exhibited anticarcinogenic activities by interfering at various stages of cancer through various mechanisms including cellular proliferation, differentiation, apoptosis, angiogenesis, and metastasis [230]. We have a rich historical record from ancient physicians about the use of natural product medicines alone and in combination, which might provide important hints for inventing new drugs. Nowadays, many anticancer drugs available in the market are natural product phytochemicals or their derivatives [231] and some are under clinical trials [232].

**Figure 9:**
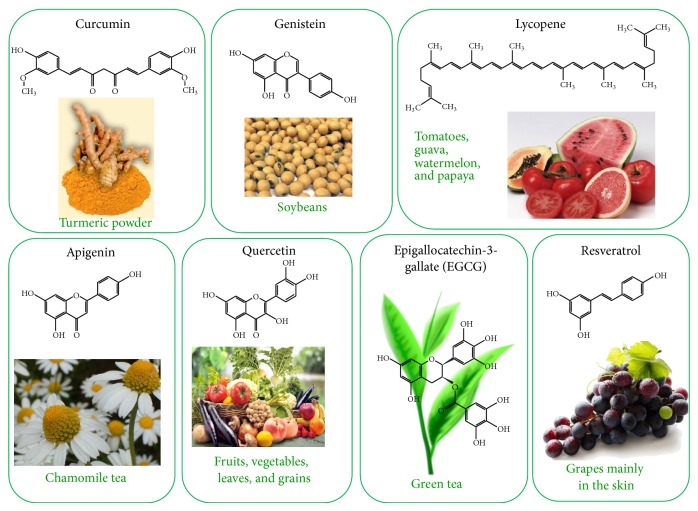
Natural product derived phytochemicals with anticancer activity perturbing IGF signalling pathways.

The natural products including curcumin (3,3′-diindolylmethane (DIM)), isoflavone genistein (indole-3-carbinol (I3C)), epigallocatechin-3-gallate (EGCG), resveratrol, lycopene, and apigenin have been recognized as cancer chemopreventive agents (Figure 9) because of their anticarcinogenic activity [233, 234]. The in vitro and in vivo studies have demonstrated that these natural products have inhibitory effects on various human and animal cancers [235–239]; therefore, many researchers have focused on interpreting the molecular mechanisms and identifying the targets of action of these natural products. The various natural products perturbing IGF signalling pathways and their mechanism of actions have been summarised in Table 7. The understanding of molecular mechanism of natural product derived phytochemical against a specific cancer type will lead to the development of novel anticancer drugs.

## 7. Future Perspectives: Challenges and Opportunities for Novel IGF Therapies

The success of targeted therapies for cancer is undisputed; strong preclinical evidence and on-going clinical trials of some of the drugs chemical molecules, antibodies, antisense technology, si-RNA therapy against members of the IGF-axis-IGF ligands, IGFBPs, and IGF-Rs have resulted in the approval of several new agents for cancer treatment. Not only targeting of these by single substances but also the approaches of cotargeting strategies seem to be a very promising avenue with more and more studies directed in this approach to solve the complications which come across while targeting specific molecules involved in cancer pathways.

Targeting IGF ligands seems to be problematic since the IGF mediated signalling has important roles in regulating cellular proliferation and apoptosis (role as circulating hormone and a tissue growth factor) apart from their increased levels in various cancers. Another important factor to bear in mind is that higher levels of IGFBPs might increase IGF-1 concentration by increasing its circulating half-life, and this may not possibly lead to increase in receptor activation at the tissue level and the link between higher IGF levels and neoplasm seems to be unclear here.

Another approach is to target IGFBPs in a way which sequesters more and more IGFs, thereby downregulating the IGF mediated signalling in cancer pathway. Since IGFBPs are further regulated by IGFBP proteases, developing mutants which lack proteolytic cleavage sites for these proteases can pave a way for strong interaction between IGF and IGFBPs. A recent study in this regard showed that novel, modified IGFBP-2 proteins (protease resistant alone or also lacked the ability to bind extracellular matrix) sequestered both the IGFs and thereby was able to inhibit tumour growth. These modified IGFBPs were found to do so by inhibition of angiogenesis both in vitro and in vivo [84]. Apart from IGF-dependent (proteolysis) activities, IGFBPs also have IGF-independent activities in relation to cancer; mutants lacking both proteolysis and matrix-binding activities may be effective for the treatment of cancers in the future.

While IGF receptors seem to be the most favourite targets in the IGF-axis in relation to cancer, the drawbacks and challenges in achieving this seem to add further complexity because of the cross talks between IGF-R mediated pathways and other growth mediated pathways in cells. Though various TKIs against IGF-1Rs seem to be in clinical trial, specificity and concentrations can be well documented in vitro while their extent of in vivo roles seems to be a question mark considering the variation in concentration among different tissues and toxicity could be another issue. Anti-IGFR antibodies are advantageous over TKIs in this regard while blockage of IGFRs may pressurize the tumour cells to compensate for blockade by increased signalling through alternate receptors (e.g., EGFRs). In some instances, IGF-2 action via the IR-A also promotes resistance to anti-IGF-1R inhibitors. Thus, specific therapeutic combinations can be an answer to this problem.

## Figures and Tables

**Table 1:** IGF-1 transgenic mice with tissue-specific IGF-1 overexpression.

Organs	IGF-1 action	Promoter	Reference
Brain	Increased brain size, characterized by increased neuron number.	M IGF-2 5′ flanking region	[35, 36, 181, 182]

Bone	Increased trabecular bone.	Bovine osteocalcin	[37]

Heart	Increased myocyte proliferation.	r *α* myosin heavy chain	[38]

Muscle: skeletal	Stimulates differentiation and myofibril hypertrophy.	Avian skeletal *α* actin	[39]

Muscle: smooth	Smooth muscle hyperplasia in many flanking fragments organs/tissues. Increased vascular contractility. Enhanced neointimal formation after injury.	r smooth muscle *α* actin (mSMA)	[183–185]

Ovary	Increased testosterone and cyst.	m LH receptor	[186]

Prostate	Epithelial neoplasia.	Bovine keratin-5	[187]

Thyroid	When the IGF-1R is also overexpressed, there is a decreased TSH requirement and goiter.	Bovine thyroglobulin	[188]

**Table 2:** Human insulin-like growth factor binding proteins.

IGFBPs	Mass (kDa)	Source of purification	Relative binding affinity for IGFs
IGFBP1	25.0	Amniotic fluid, placenta	IGFI = IGFII
IGFBP-2	31.3	BRL-3A and MDBK cells, human serum	IGFII > IGFI
IGFBP-3	28.7	Plasma	IGFI = IGFII
IGFBP-4	25.9	Human osteosarcomas, prostatic carcinoma, colon carcinoma, and glioblastoma	IGFI = IGFII
IGFBP-5	28.5	C2 myoblasts conditioned media, human bone	IGFI = IGFII
IGFBP-6	22.8	Cerebrospinal fluid, human serum	IGFII > IGFI

**Table 3:** Consequences of inhibiting IGFBP activity and cancer.

IGFBPs	Expression	Results of inhibiting IGFBP activity	Reference
IGFBP-1	Liver	It can induce or inhibit the IGF actions in many types of cells. As an example of the inhibiting activity of IGFBP-1, it inhibited IGF-I-induced growth in MCF-7 breast cancer cells.	[30, 157]

IGFBP-2	Liver, adipocytes, reproductive system, and central nervous system	IGFBP-2 level changes were associated with the development of different types of cancer including breast and prostate cancer. In prostate cancer, high level of serum IGFBP-2 was associated with low grade prostate cancer.	[189, 190]

IGFBP-3	Circulating carrier protein, expressed in many tissues	IGFBP-3 plays important role in different types of human cancers. IGFBP-3 can induce apoptosis by increasing the ratio of proapoptotic to antiapoptotic proteins in breast cancer cells.	[191]

IGFBP-4	Liver, bone tissue, and muscles	IGFBP-4 showed a strong inhibitory effect on IGF-1 by preventing the activation of the IGF-1R, when the IGFBP-4 is found in the tissue. Conversely, intravenous administration of IGFBP-4, in the presence of a protease, will promote cellular proliferation.	[79, 192–195]

IGFBP-5	Mammary glands	In breast cancer, IGFBP-5 induced apoptosis and inhibited cellular differentiation in an IGF-dependent manner.	[196, 197]

IGFBP-6	Epithelial layer of human bronchial organ	It can inhibit IGF-2 activity mediated through the IGF-1R, including proliferation, differentiation, migration, and survival in different cell lines.	[198, 199]

**Table 4:** Summary of IGFBP proteases and their proteolytic cleavage sites.

Proteolytic cleavage sites	IGFBP protease	Reference
IGFBP-2		
Met166-Gly167, Lys168-Gly169, Tyr103-Gly104, Leu152-Ala153, Arg156-Glu157, Gln165-Met166, Thr205-Met206, Arg287-Met288	Unknown protease in hemofiltrate	[80]
Leu3-Phe4, Lys168-Gly169, Lys181-Leu182	Unknown in milk	[200]
Arg164-Gln165	Human kallikrein-2	[201]
Leu152, Gly175-Leu176, Lys181-Leu182	Matrix metalloproteinase-7	[202]
Gln165-Met166	PAPP-A	[203]
His165-Arg166	Calpain	[204]

IGFBP-3		
Arg97-Ala98, Lys160-Val161	Plasmin	[205]
Arg95-Leu96, Lys160-Val16	Plasmin	[206]
Arg97-Ala98, Arg206-Gly207	Thrombin	[205]
Arg97-Ala98, Lys149-Lys150, Lys150-Gly151, Lys154-Asp155	Serum	[205]
Arg97-Ala98, Arg132-Val133, Tyr159-Lys160, Phe173-Ser174, Arg179-Glu180	Seminal plasma PSA	[157]
Arg97-Ala98, His131-Arg132, Tyr159-Lys160	Urinary PSA	[207]
Arg97-Ala98	Cysteine protease from MCF-7 cells	[208]
Tyr99-Leu100, Leu96-Arg97, Leu141-His142	MMP-1, MMP-2	[176]
Tyr99-Leu100, Asn109-Ala110, Glu176-Ser177	MMP-3	[176]

IGFBP-4		
Lys120-His121	Calcium-dependent serine protease from smooth muscle cells	[193, 209, 210]
Met135-Lys136	PAPP-A	[211, 212]

IGFBP-5		
Arg138-Arg139	Serine protease from smooth muscle cells	[213]
Ser143-Lys144 (secondary cleavage site), Ser143-Lys144	PAPP-A2	[214]
Gln142-Ser-143	PAPPA	[214]
Lys120-His121, Arg156-Ile157, Arg192-Ala193	Thrombin	[215]

**Table 5:** Few examples of small molecule TKIs (tyrosine kinase inhibitors) directed against IGF receptors.

Small molecule inhibitor	Mode of action	Effects	Reference
NVP-AEW541 NVP-AEW54 in combination with gemcitabine	Kinase inhibition	Antineoplastic, tumour regression and inhibition of metastasis	[216, 217]

Picropodophyllin (PPP)	Against autophosphorylation at the substrate level	Inhibition and downregulation of IGF-1R	[218–221]

BMS-554417	ATP-competitive, dual kinase inhibition	Antiproliferative activity	[222]

INSM-18	Reversible ATP-competitive	Inhibitor of transcription (blocking also cdc2, survivin, and VEGF)	[223]

OSI-906	Reversible ATP-competitive	Derived from compound-1, also known as PQIP	[223]

XL-228 (XL-2280)		Inhibits bcr-abl, scr, and IGF-1R	[224]

BVP-51004 Biovitrum (Cyclolignan PPP)	Non-ATP-competitive	Causes IGF-1R downregulation, probably through the induction of ubiquitination.	[223]

**Table 6:** Few examples of anti-IGF-RI monoclonal antibodies (MAbs) [223].

Monoclonal antibody	Class	Clinical information
CP-751,871	Fully human IgG2 mab	Ewing's sarcoma family of tumours, breast cancer, single agent in metastatic CRC

IMC-A12	Fully human IgG1 mab	Ewing's sarcoma family of tumours CRC and H&N cancer

R1507	Fully human IgG1 mab previously known as RO4858696	Pediatric patients and sarcomas.

AMG-479	Fully human mab	Ewing's sarcoma family of tumours, pancreatic cancer

SCH-717454	Fully human mab previously known as 19D12 (Medarex)	Colorectal cancer (CRC)

AVE-1642	Humanized mab	Previously known as EM164 (ImmunoGen)

MK-0646	Fabre Humanized mab Previously known as A2CHM, F50035, 7C10, or 7H2HM	Colorectal cancer (CRC)

BIIB022	Fully human nonglycosylated IgG4.P antibody	Devoid of Fc-effector function to eliminate potential Fc mediated toxicity to the normal vital organs.

**Table 7:** Natural products perturbing IGF signalling pathways.

Active phytochemicals	Natural source	Mode of action	Molecular target
Curcumin [225, 226]	*Curcuma longa (turmeric powder) *	Antiproliferation, anticarcinogenesis, cell cycle arrest, apoptosis, and antiangiogenesis	IGF-1R

Genistein [226]	Soybeans and soy products, red clover (*Trifolium pratense*), and sicilian pistachio (*Pistacia vera*)	Antioxidant, antiproliferation, antiproliferation, anticarcinogenesis, cell cycle arrest, apoptosis, antiangiogenesis, and anti-inflammation	IGF-1R

Lycopene [226]	Tomatoes, guava, rosehip, watermelon, papaya, apricot, and pink grapefruit; most abundant in red tomatoes	Antioxidant, antiproliferation (growth inhibition, cell cycle arrest, and apoptosis), antiangiogenesis, anti-inflammation, and immunomodulator	IGFBP-3

Apigenin [227]	Fruits and vegetables, including oranges, grapefruits, parsley, celery, onions, wheat sprouts, cereals of millet and wheat, and in some seasonings, such as coriander, marjoram, oregano, rosemary, tarragon, and chamomile tea	Inhibit cellular proliferation, suppress tumorigenesis and angiogenesis, and induce apoptosis	IGF axis and its intracellular signalling in prostate cancer

Quercetin [228]	Fruits, vegetables, leaves, and grains	Inhibits the proliferation and induces apoptosis of cancer cells	IGFIR

Epigallocatechin-3-gallate [229]	Green tea	Inhibits angiogenesis	Inhibitory effects on IGF-I-induced VEGF expression

Resveratrol [225]	Grapes (mainly in the skin), mulberries, peanuts, vines, and pines	Antioxidant, antiproliferation, anticarcinogenesis, cell cycle arrest, apoptosis, antiangiogenesis, and anti-inflammation	Suppression of IGF-1R/Akt/Wnt signalling pathways
